# Supplementary material for: Predicting SARS-CoV-2 Variant Spread in a Completely Seropositive Population Using Semi-Quantitative Antibody Measurements in Blood Donors
Source: Vaccines (Basel). 2022 Aug 31;10(9):1437. doi: 10.3390/vaccines10091437 (PMC9501043; doi:10.3390/vaccines10091437)
Supplement: Supplementary file 1 [file vaccines-10-01437-s001.zip › vaccines-1846756-supplementary.pdf]

Supplemental material

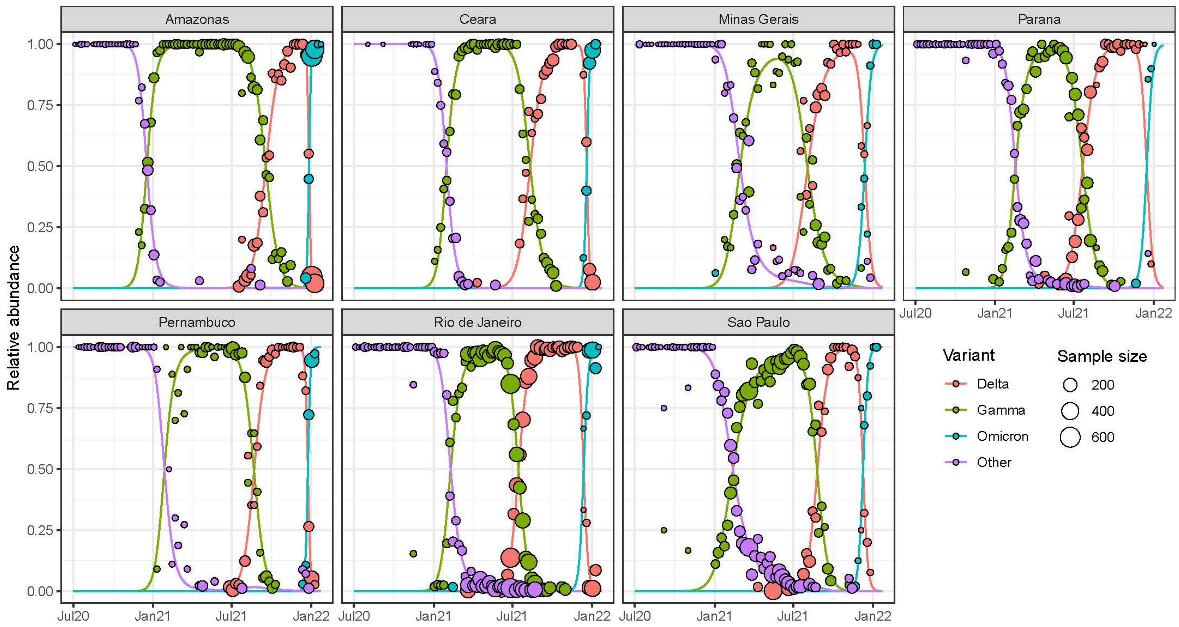

Figure S1. The metadata for all SARS-CoV-2 sequences deposited on GISAID between Jul 2020 and Jan 2022 (https://www.gisaid.org/) were downloaded. The lines show the predictions of a multinomial model fit using the nnet package in R. “Other” refers principal to wild type virus and P.2.

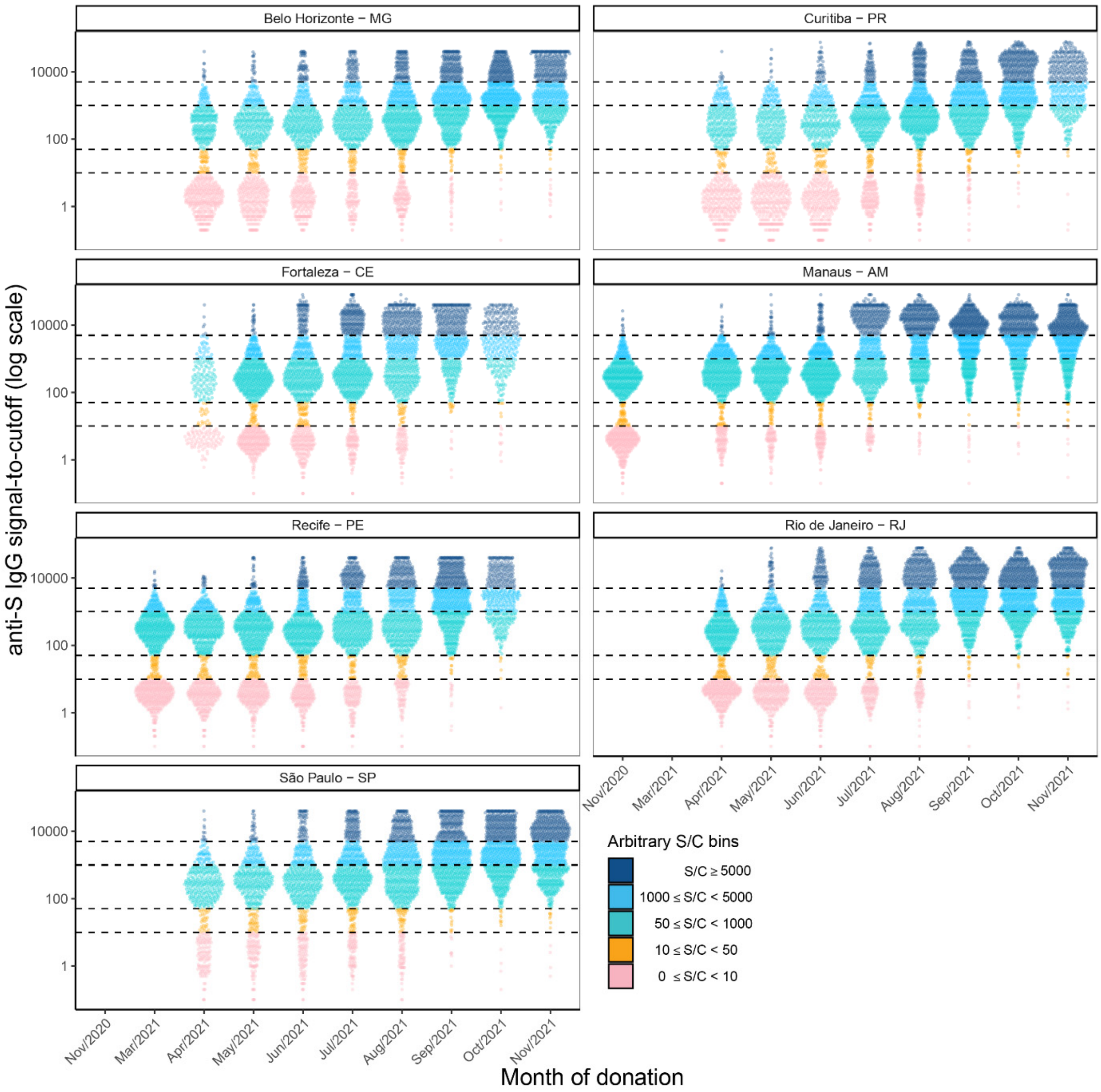

Figure S2. Raw signal to cut-off readings for each month blood donor sample across the seven Brazilian state capital cities. A threshold of 50 S/C is defined as a positive assay, other thresholds are arbitrary and shown to aid visualization.

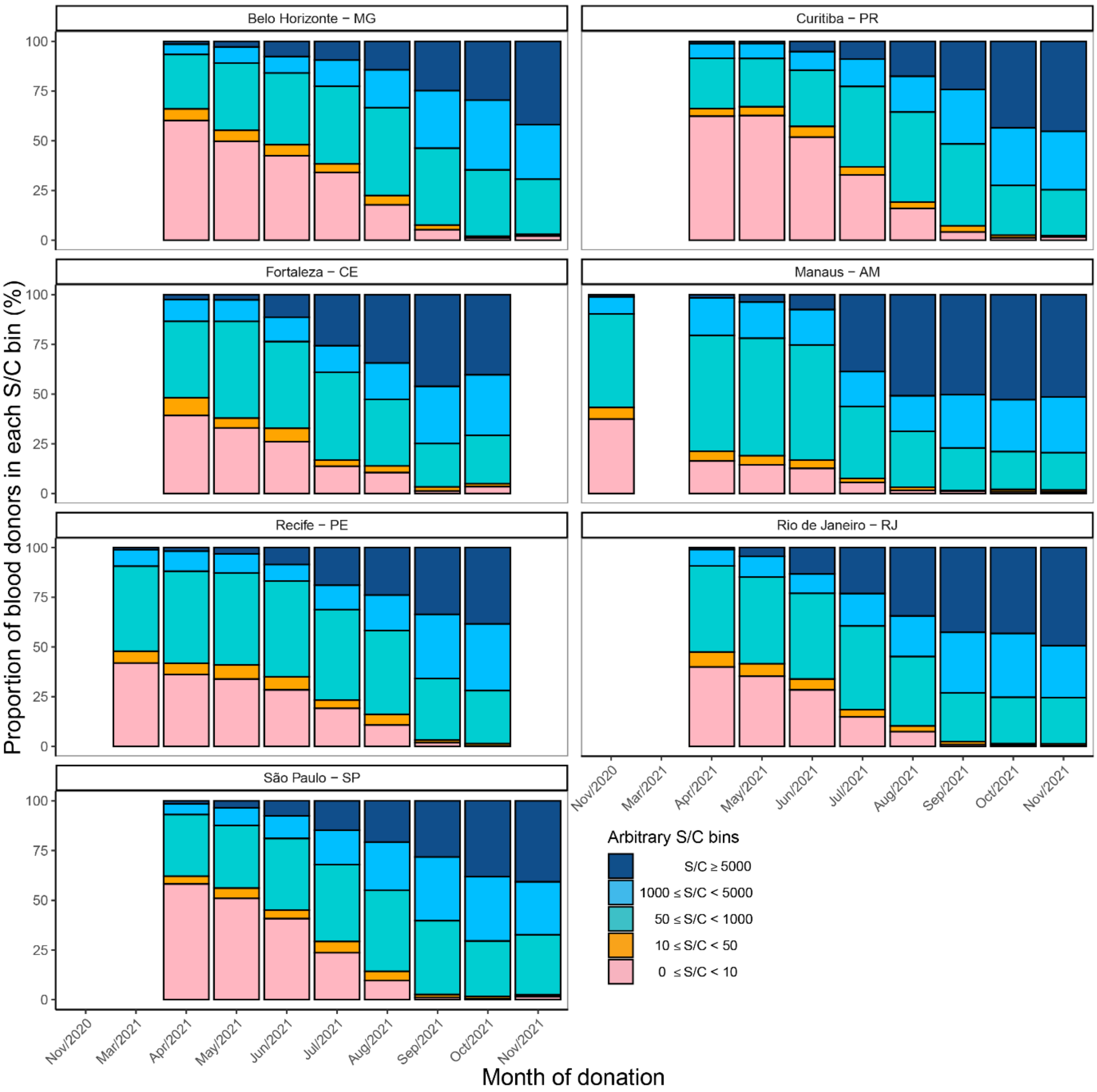

Figure S3. Proportion of blood donor samples falling in arbitrary S/C bins each month across seven Brazilian state capitals.

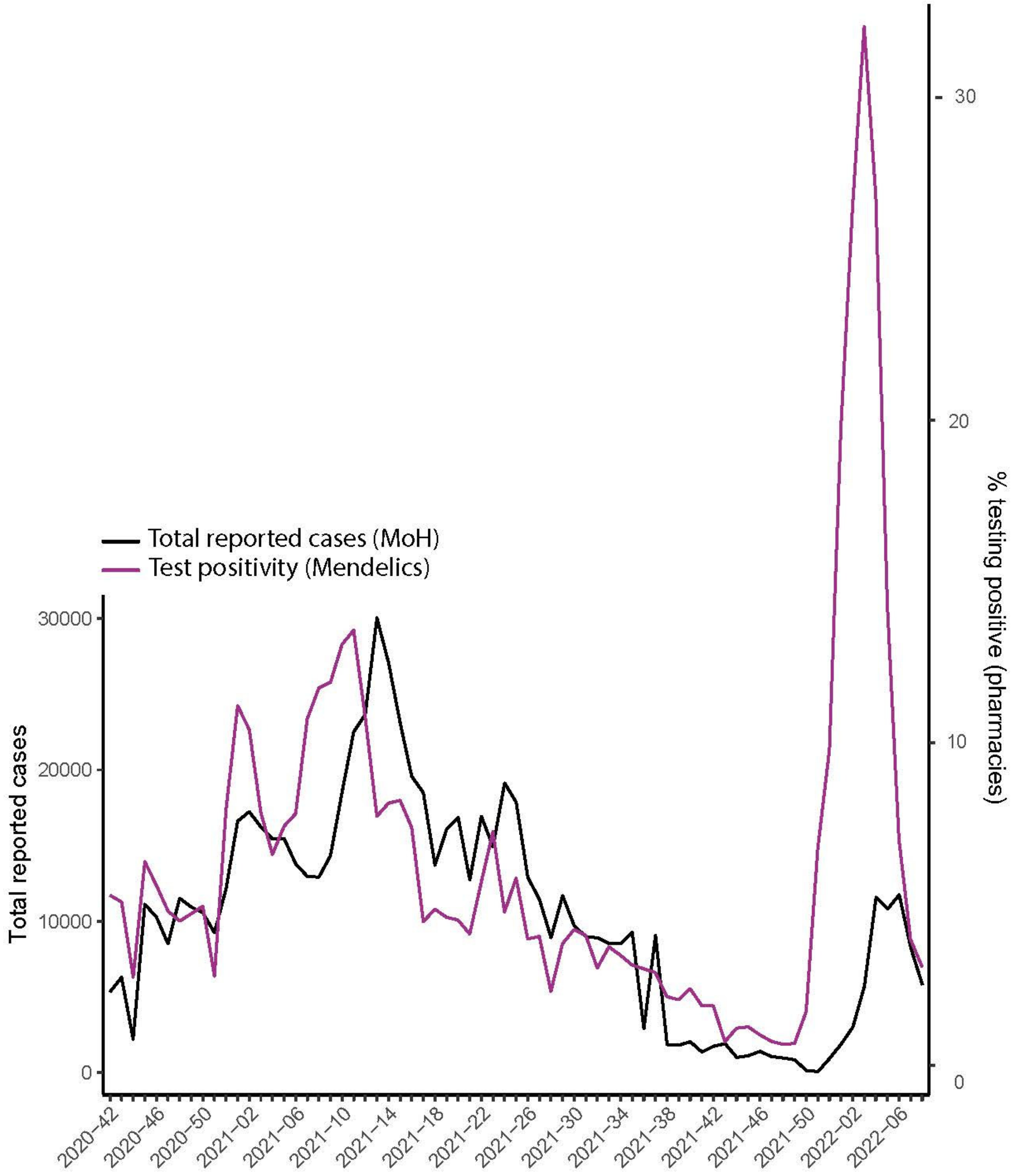

Figure S4. Black line (LHS y-axis) **-** total number of reported cases in Sao Paulo city (https://www.covid.saude.br/) shown per week of reporting. Purple line (RHS y-axis) shows the % positivity of tests administered in pharmacies in Sao Paulo by Mendelics (https://mendelics.com.br/).
